# Evaluation of a novel PCR-based assay for the detection of *Candida auris* colonization

**DOI:** 10.1128/spectrum.02336-25

**Published:** 2026-01-09

**Authors:** Jesse Leonard, Alicia Amamoto, Monica Bates, Anh T. Tran, Pooja Ghatbale, Ahnika Kline, David T. Pride

**Affiliations:** 1 Department of Pathology, University of California, San Diego 8784, La Jolla, California, USA; 2 Department of Medicine, University of California, San Diego 8784, La Jolla, California, USA; The George Washington University School of Medicine, Washington, DC, USA

**Keywords:** *Candida auris*, colonization, real-time PCR, infection prevention, Simplexa direct

## Abstract

*Candida auris* is an emerging fungal pathogen linked to healthcare-associated infections, necessitating rapid and accurate detection for effective infection control. This study evaluated the analytical and clinical performance of the Diasorin Simplexa *C. auris* Direct IVD assay compared to traditional culture as the reference standard and also to two lab-developed tests (LDTs) already validated in our laboratory: one using DSQ Alert primers/probes on the Roche Cobas 5800 and another on the DiaSorin LIAISON MDX platform. Clinical accuracy was assessed using deidentified residual axillary/groin surveillance specimens from 20 culture-positive and 20 culture-negative patients. Analytical specificity was tested against 14 microbial species commonly found in the axilla/groin. Analytical sensitivity was determined using ZeptoMetrix *C. auris* strain Z485. Specimen stability was examined at various temperatures. Environmental swabbing was also conducted before and after sample handling. The Diasorin Simplexa *C. auris* IVD assay demonstrated 100% sensitivity and 90% specificity relative to culture and showed 100% positive agreement with both LDTs and 100% negative percent agreement with the DSQ Alert LDT. The assay also achieved 100% analytical specificity, with no cross-reactivity observed, and an analytical sensitivity of 702 CFU/mL. Specimen integrity remained stable for up to 30 days under all storage conditions. No environmental contamination was detected. The DiaSorin Simplexa *C. auris* IVD assay demonstrated strong analytical and clinical performance while offering advantages in speed, ease of use, and flexible batch size. It may serve as a practical solution for institutions seeking timely *C. auris* screening in support of infection prevention protocols.

**IMPORTANCE:**

*Candida auris* is an important yeast notable for its ability to easily colonize people as well as its drug resistance. Colonized patients who become immunocompromised may ultimately become infected with *C. auris*, without many options for treatment. Many health care facilities mandate screening patients for colonization with this yeast to help prevent transmission. This work evaluates the recently FDA approved Diasorin Simplexa *C. auris* PCR based screening assay and compares its performance to cultures, as well as two existing lab-developed assays. It is the first assay performance evaluation outside of the FDA submission.

## INTRODUCTION

*Candida auris* is an emerging fungal pathogen first described in 2009 after being isolated from a patient’s ear canal in Japan (1). The first case of *C. auris* in the United States occurred in 2013, and since then, the number of annual cases has risen dramatically (2). Between 2019 and 2022, the number of clinical *C. auris* and colonization cases increased by 214% and 339%, respectively (3). In 2020, at the onset of the COVID-19 pandemic, the Centers for Disease Control and Prevention (CDC) reported a concerning spread of *C. auris* across healthcare facilities in the United States. By 2021, the number of isolates exhibiting resistance to echinocandins, the standard treatment for *C. auris* infections, had tripled (4).

Since then, *C. auris* has become a global concern, with cases in at least 50 countries on six continents (5, 6). In response to this threat, the World Health Organization recently classified *C. auris* as a critical fungal pathogen on its Fungal Priority Pathogens List. Additionally, the CDC has acknowledged *C. auris* as a public health threat, requiring urgent and aggressive intervention. This classification is due to the organism’s ability to cause invasive infections with high mortality rates, its potential for widespread outbreaks in healthcare settings, its capacity to asymptomatically colonize human skin, its intrinsic resistance to most available antifungal treatments and common disinfectants, and the challenges it presents in accurate identification and characterization using traditional laboratory methods (7, 8).

Not only is it difficult to distinguish *C. auris* from other *Candida* species using conventional laboratory methods, but many studies have also reported that biochemical-based tests misidentify *C. auris* as other *Candida* species or even genera (9–11). This misidentification can lead to misdiagnosis, inappropriate management, and missed opportunities for infection control. Currently, the CDC recommends screening patients at risk for *C. auris* colonization, including those with an epidemiological link to a colonized patient or facility, and healthcare facilities both within and outside the United States located in high-prevalence areas. Patients with risk factors for acquiring *C. auris*, such as mechanical ventilation, in-dwelling medical devices, receipt of high acuity medical care, frequent or long healthcare stays, infection, or colonization with other multidrug-resistant organisms (12). Current guidance suggests swabbing the axilla and groin though recent data suggest improved detection with the inclusion of anterior nares and hands (13, 14).

The aforementioned reasons are justification for why rapid and accurate detection of *C. auris* colonization is critical for the initiation of proper infection prevention measures and contact precautions to limit the spread of this fungal pathogen in healthcare settings. Recognizing the urgency of early detection, this study aims to assess a diagnostic tool that may improve the detection and management of *C. auris*. This study was designed to assess the performance of the Simplexa *C. auris* Direct Assay, a real-time polymerase chain reaction (RT-PCR) assay intended for use on the LIAISON MDX (15), by examining clinical accuracy, analytical sensitivity, and cross-reactivity during implementation in a clinical laboratory. This study also examined the potential impact of proper handling on environmental contamination with *C. auris*.

## MATERIALS AND METHODS

### Diasorin *C. auris* IVD

The Simplexa *C. auris* Direct is an FDA-approved real-time PCR assay designed for the direct amplification and qualitative detection of *Candida auris* DNA from composite axilla/groin swabs. This system utilizes the LIAISON MDX platform (integrated with LIAISON MDX Studio Software), the Direct Amplification Disc, the Simplexa *C. auris* Direct Assay, the Simplexa *C. auris* Sample Prep Solution, and essential laboratory consumables. The assay employs specific primers and FAM-labeled fluorescent probes to amplify both *C. auris* DNA and the assay internal control, ensuring reliability of its results. The assay targets the *C. auris* ITS 2 rRNA gene. A *C. auris* DNA positive control is included to aid in the detection of potential PCR failures or inhibition. A “No Template Control” (NTC) consisting of fresh Liquid Amies media and a positive *C. auris* DNA control were both run each day before testing to verify assay performance in the selected specimen media (13). The Simplexa *C. auris* Direct assay was performed according to the manufacturer’s instructions for use. Briefly, 50 µL of reaction mix was loaded onto the Direct Amplification Disc. Next, 50 µL of the patient sample was transferred to the Sample Prep Solution vial and mixed thoroughly by pipetting up and down, then 50 µL of the mixed sample was loaded onto the Direct Amplification Disc. The reaction wedge was sealed using the disc’s adhesive foil prior to being run on the LIAISON MDX. Results were produced approximately 105 min later.

### Diasorin *C. auris* LDT

The *C. auris* Primer Pair LDT utilizes Diasorin Molecular’s *C. auris* Primer Pair, Fungal Lysis Solution and Simplexa Extraction and Amplification primers and control, with fluorescent probe technology and real-time PCR amplification and detection on the LIAISON MDX system. Each probe molecule is affixed with a fluorophore and a quencher. The *C. auris* primer pair is an analyte specific reagent (ASR) introduced in 2019 for amplifying a well-conserved region of the ITS2 region in the rRNA gene for *Candida auris* with a FAM labeled probe, forward primer, and reverse primer in Tris-EDTA buffer. First, 5 µL of Fungal Lysis Buffer is added to 50 µL of patient sample, and then the mix is incubated at 60°C for 30 min. During the incubation period, a master mix is made using *C. auris* Primer Pair solution, SEAC (IC) Primer Pair control, SEAC Control DNA (IC), and Nuclease-free water. After the incubation period, exactly 8 µL of master mix is then added to a reaction well on a 96-well disc, followed by 2 µL of fungal lysate. Each reaction wedge is then covered with Universal Disc Cover Tape prior to being run on the LIAISON MDX for 45 cycles. Results were produced approximately 73 min later.

### DSQ *C. auris* LDT

The DSQ Alert *Candida auris* Assay is a real-time PCR LDT performed on the Cobas 5800 System. Specimens were processed using the Cobas omni Utility Channel Reagent Kit, which includes lysis, magnetic particle-based purification, and the elution of nucleic acids. The assay targets a 124-bp region of the ITS2 rRNA gene using specific primers and FAM-labeled probes provided in the DSQ Alert Primer and Probe Mixes kit purchased from ELITechGroup. Myriad consumables were replaced prior to each run, including tip racks, analyte-specific reagents, negative control racks, processing, liquid waste, and amplification plates, alongside wash, lysis, and diluent reagents. An internal control was co-extracted and co-amplified to monitor assay performance. Amplification and detection occurred in a closed system, using fluorescent signals generated during probe cleavage. The master mix includes dUTP and AmpErase enzymes to prevent carryover contamination. The cycling parameters of the assay include 45 cycles of denaturation and annealing steps, lasting 5 and 25 s, respectively, at temperatures of 91°C and 58°C. The system automated all sample preparation, PCR amplification, detection, and data analysis, with each 24-well plate accommodating 350 µL from up to 22 samples alongside an external control and an NTC. Results were produced approximately 2.5 h after fully loading the instrument.

### Clinical accuracy

To evaluate the clinical accuracy of the Diasorin *C. auris* IVD assay, a total of 40 previously characterized composite axilla/groin swab specimens were tested, including 20 positive and 20 negative specimens. Swabs were collected using either BD ESwab or Copan ESwab in 1 mL liquid amies. Each positive sample was retrospectively obtained from frozen specimens. All clinical specimens were collected from patients with risk factors for *C. auris* colonization. Specimens were de-identified and assigned unique identification numbers to ensure the operator was blinded to both the subject identity and comparator results before testing. Culture served as the primary reference method for the primary analysis. A secondary analysis was also performed comparing different PCR methods to each other.

### Culture comparator

Retrospective specimens were utilized in this study that had been stored at −70°C. The retrospective specimens were thawed and plated onto chromogenic agar that allows for the growth and differentiation of *C. auris* as well as other *Candida* species (Hardy Chromagar, Cat# G343) using 10 µL of the specimen and streaked for isolation and incubated at 37°C. Candidate colonies exhibiting color changes characteristic of *C. auris* were confirmed by mass spectrometry using the Bruker MALDI-ToF (Library version IVD MBT Compass 4.1.100). All Diasorin *C. auris* IVD assay specimens with an invalid result or error were re-tested once to obtain a valid result. For specimens that were Diasorin *C. auris* IVD PCR-positive but culture-negative, culture plates were incubated for 2 additional days to ensure the lack of detectable growth.

### Analytical sensitivity/limit of detection

To evaluate the analytical sensitivity of the Diasorin *C. auris* IVD assay, experiments were performed to identify the assay’s limit of detection (LoD) in a negative bilateral axilla/groin swab matrix. Analytical sensitivity was determined by creating a series of serial dilutions using a commercial *C. auris* stock (Zeptometrix Cat# 0804386) that was quantified to contain 10^9^ CFU/mL by viable plate count. This stock was diluted from 10^9^ to 10^2^ CFU/mL, and each dilution magnitude was tested in triplicate. Then, 20 replicates were performed at the lowest concentration detected to determine whether that concentration represented the LoD.

### Analytical specificity/cross-reactivity

The analytical specificity, or cross-reactivity, of the Diasorin *C. auris* IVD assay was evaluated using a panel of 14 organisms that are either closely related to *C. auris* or anticipated to be present in the axilla or groin regions. The organisms representing bacteria and fungi are *A. fumigatus*, *C. albicans*, *C. glabrata*, *C. parapsilosis*, *C. dubliniensis, C. lusitaniae*, *C. kruseii*, *C. guillermondii, C. tropicalis, C. neoformans, E. coli*, *S. cerevisiae, S. epidermidis*, and *T. rubrum*. Quantified Zeptometrix stocks were diluted to clinically relevant concentrations (10^5^–10^6^ CFU/mL) in Liquid Amies medium and then tested in triplicate.

### Stability

The stability of bilateral axilla/groin swabs in Liquid Amies media (BD ESwab) and its potential impact on the performance of the Diasorin *C. auris* IVD Assay was also evaluated. In order to examine specimen stability, a *C. auris* standard contrived to 5× LoD was prepared in triplicate and aliquoted for various time points. The aliquots were stored at room temperature (22–25°C), refrigerated (2–8 °C), and frozen (<−70°C) and then tested in triplicate at the time points outlined (Table 1).

**TABLE 1:** Test time points and storage conditions for stability testing

Storage condition	Test time points
Room temperature (22– 25°C)	0 h, 48 h, 72 h
Refrigerated (2–8°C)	0 h, 72 h, 7 days
Frozen (<−70°C)	0 h, 30 days

### Environmental swab testing

The sites designated for swab testing were the inside of the instrument and the outside of the instrument. The areas swabbed outside of the instrument include the instrument’s lid, open button, logos, mouse keys and body, laptop keys, body, and trackpad, bench space, pipettes, pipette boxes, and the surface of the biosafety cabinet. The inside of the instrument was also swabbed in areas like the central testing platform, under the removable magnetic ring, the ring itself, as well as all openings, holes, and crevices within. Swabs were collected using either BD ESwab or Copan ESwab in 1 mL Liquid Amies. Swab specimens were then tested on the Direct Amplification Disc as per the Simplexa *C. auris* Direct Assay’s instructions for use.

### Statistical analysis

Positive Percent Agreement (PPA), Negative Percent Agreement (NPA), Probit, Cohen’s kappa, diagnostic accuracy, and two-sided (upper/lower) 95% confidence intervals (CI) were calculated using Microsoft Office Excel 365 MSO software (Microsoft, Redmond, WA) and Prism GraphPad software (GraphPad Software Inc., San Diego, CA, USA). The PPA was calculated as TP/(TP + FN) × 100, the NPA was calculated as TN/(TN + FP) × 100, where TP is true-positive results, FN is false-negative results, TN is true-negative results, and FP is false-positive results. Probit analysis was used for the CFU/mL determination of the analytical sensitivity study. Diagnostic accuracy was calculated as [(TP + TN)/(TN + FP + FN + TP)]. Cohen’s kappa values (*κ*) were calculated as a measure of the overall agreement, categorized as almost perfect (>0.90), strong (0.8 to 0.9), moderate (0.6 to 0.79), weak (0.4 to 0.59), minimal (0.21 to 0.39), or none (0–0.20).

## RESULTS

### Comparison of assay accuracy

Specimens selected for testing using the Diasorin *C. auris* IVD assay on the LIAISON MDX and for comparator *C. auris* culture were 40 previously characterized *C. auris* specimens. Specimens were previously determined positive or negative using the Diasorin *C. auris* LDT on the LIAISON MDX and/or the DSQ *C. auris* LDT on the Cobas 5800 with testing platforms evenly distributed across 20 previously positive *C. auris* specimens and 20 previously negative *C. auris* specimens. The sensitivity of the Diasorin *C. auris* IVD Assay compared to the gold-standard reference method of culture followed by MALDI identification was 100% (95% CI 0.82–1), and the specificity was 90% (95% CI 0.72–0.98) (Table 2). There were two retrospective specimens that were detected by the Simplexa *C. auris* Direct IVD assay but exhibited no growth in culture. Both of these specimens were also detected by both PCR-based LDT methods. The diagnostic accuracy of the Simplexa *C. auris* Direct Assay compared to the culture comparator was 95% (95% CI 0.83–0.99), showing a high rate of accuracy. The Cohen’s kappa value was 0.9 (95% CI 0.76–1), demonstrating strong agreement.

**TABLE 2:** Diasorin *C. auris* IVD vs culture^*a*^

Diasorin *C. auris* IVD	Reference method (culture + MALDI)			± 95% CI			
	Positive	Negative	Total	Sensitivity	Specificity	Accuracy	Kappa (*κ*)^*b*^
Positive	18	2	20	100%(0.82–1)	90% (0.72–0.98)	95% (0.83–0.99)	0.9 (0.76–1)
Negative	0	20	20				
Total	18	22	40				

^*a*^ The clinical performance of the *C. auris* IVD assay compared to culture and MALDI-ToF was evaluated. Sensitivity, specificity, diagnostic accuracy, and Cohen’s kappa values with 95% CI are shown.

^*b*^ κ = inter-assay agreement; 95% CI shown.

The clinical accuracy of the Diasorin *C. auris* IVD Assay was also compared to the two in-house *C. auris* LDT methods (Table 3). The Diasorin *C. auris* IVD assay demonstrated perfect agreement with the DSQ Alert LDT assay across both positive and negative samples with a PPA of 100% (20/20, 95% CI 0.83–1) and NPA of 100% (20/20, 95% CI 0.83–1). The corresponding Cohen’s kappa value for this assay comparison was 1.00, indicating perfect overall agreement. For the comparison between the Diasorin *C. auris* IVD and the Diasorin *C. auris* LDT, only positive samples were available. The PPA between these two assays was 100% (13/13, 95% CI 0.77–1). Both NPA and Cohen’s kappa values could not be calculated for this comparison because the negative retrospective specimens used in this study were not run on the Diasorin *C. auris* LDT, making chance-corrected agreement between these two assays uninterpretable. However, the consistent positive agreement observed across all assay comparisons provides strong initial evidence of consistent performance across assay platforms for *C. auris* positive samples and the Direct IVD assay’s diagnostic concordance.

**TABLE 3:** Diasorin *C. auris* IVD vs LDT^*a*^

Diasorin *C. auris* IVD	DSQ *C. auris* LDT			± 95% CI^*b*^		
	Positive	Negative	Total	PPA	NPA	Kappa (*κ*)
Positive	20	0	20	100%(0.83–1)	100%(0.83–1)	1 (0.99–1)
Negative	0	20	20			
Total	20	20	40			

Diasorin *C. auris* IVD	Diasorin *C. auris* LDT			± 95% CI^*b*^		
	Positive	Negative	Total	PPA	NPA	Kappa (*κ*)
Positive	13	0	0	100% (0.77 – 1)	N/A^*c*^	Not defined
Negative	0	0	0			
Total	0	0	13			

^*a*^ The Diasorin *C.auris* IVD agreement with the Diasorin *C. auris* LDT and DSQ *C. auris* LDTs was evaluated. PPA and NPA agreement with 95% CI are shown. NPA is not defined for the comparison between the Diasorin *C. auris* IVD and the Diasorin *C. auris* LDT due to absence of concordant negative specimens. A Cohen’s kappa value of 1 represents perfect agreement.

^*b*^ PPA, percent agreement; NPA, negative percent agreement; κ, inter-assay agreement, 95% CI shown.

^*c*^ N/A, not applicable.

### Limit of detection analysis

We next determined the limit of detection for the Diasorin *C. auris* IVD assay. We had previously determined that the LoD for the DSQ *C. auris* LDT was 100 cp/mL when run on the Cobas 5800 and that the limit was 1,000 cp/mL for the Diasorin *C. auris* LDT run on the LIAISON MDX (data not shown). In contrast to the previous LoD experiments done for our LDT assay validations, which utilized inactivated *C. auris* stock solutions quantified in copies/mL, our experiments were conducted using a live *C. auris* stock solution quantified in CFU/mL by plating and counting prior to testing in order to account for the viability of our control organism. We diluted our *C. auris* stock solution from 10^9^ CFU/mL to a clinically relevant concentration of 10^5^ CFU/mL and then tested each 10-fold dilution in triplicate until we achieved our first dropout value at 10^2^ CFU/mL. We then tested 20 separate replicates at the lowest concentration where amplification was detected in all three dilution replicates. The detection of *C. auris* in 19/20 (95%) replicates detected was considered acceptable for the limit of detection of the assay. We performed serial dilutions and testing of 20 replicates until less than 95% detection occurred. We, thus, determined the LoD of the assay to be 702 CFU/mL (Table 4). As an additional investigation, we also conducted a probit analysis using data from three independent iterations of the Diasorin *C. auris* IVD assay’s sensitivity analysis, which would refine the LoD to 317 ± 56 CFU/mL based on the detection rates across varying concentrations (data not shown).

**TABLE 4:** Analytical sensitivity (LoD)^*a*^

Concentration (CFU/mL)	CFU/reaction	Number tested	Number detected	Minimum CT	Maximum CT	% Detected
1,510	75.5	20	20	27.7	32.0	100%
702	35.1	20	20	29.3	32.8	100%
233	11.7	14	12	30.0	37.9	85%

^*a*^ The LoD was defined as the lowest concentration where ≥95% of the 20 replicates tested were positively detected. The limit of detection (LOD) for *C. auris* using the Simplexa Direct IVD assay was determined to be 702 CFU/mL. One reaction includes 50 mL of specimen.

### Analytical specificity

**Fig 1:**
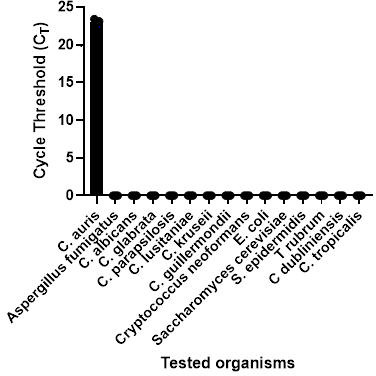
A total of 14 contrived standard solutions of clinically relevant fungi and bacteria were tested using the Simplexa *C. auris* direct assay. Zero is depicted as no amplification on this chart.

We characterized the specificity of the assay by performing the Diasorin *C. auris* IVD assay against a panel of known positive specimens containing various yeasts, molds, and bacteria. These organisms included multiple species of *Candida*, as well as *Cryptococcus, Saccharomyces, Aspergillus, Trichophyton, Escherichia,* and *Staphylococcus*. Aside from *Candida auris*, none of these organisms produced positive results on the Direct assay. These findings indicate that no cross-reactivity was observed for any of these targets, yielding an analytical specificity of 100% (Fig. 1).

### Specimen stability

**Fig 2:**
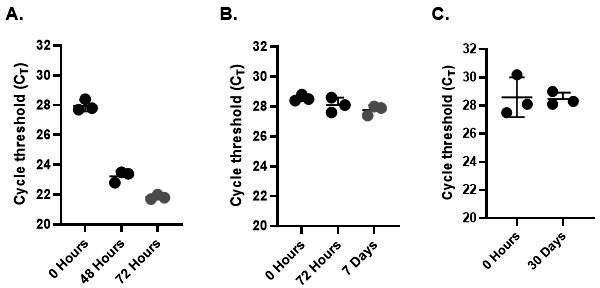
To assess specimen stability, triplicates of contrived *C. auris* samples were prepared in a negative bilateral axilla/groin swab matrix with liquid amies at 5× LOD and stored at (**A**) room temperature (22–25°C), (**B**) refrigerated (2–8°C), and (**C**) frozen at ≥ 70°C. The assay successfully identified *C. auris* under all three environmental conditions.

We evaluated the potential impact of stability of specimens stored at room temperature, refrigerated, or frozen at ≤−70°C on the performance of the Diasorin *C. auris* IVD assay. Notably, the room temperature experiments expand upon the timeframe that is currently allowed in the assay instructions for use (1). To perform this analysis, we contrived a set of specimens that were spiked with a live *C. auris* standard at 5× the LoD in a *C. auris* negative axilla/groin matrix in Liquid Amies and stored under various clinically relevant conditions for different time periods. The specimens were tested on the Simplexa *C. auris* Direct assay at the relative timepoints for each temperature condition, and the resulting CT values were recorded. We found that for all conditions over the course of the 30-day study, all expected positive results remained positive, with a trend in lower CT values over time (Fig. 2).

### Environmental colonization

Standard lab quality monitoring practices to monitor for false positivity include performance of wipe testing to monitor for environmental colonization (16). Over a 14-day period, we swabbed the interior of the LIAISON MDX instrument, the exterior of the instrument, the surfaces of the biosafety cabinet, and all tools used where the experiments were being conducted to determine whether any of those surfaces may be exposed to *C. auris* that could contaminate the experiments. The swabs were collected in a *C. auris* negative axilla/groin matrix in Liquid Amies medium and tested using the Simplexa *C. auris* Direct assay on the LIAISON MDX. Each of the tests produced negative results (Table 5), indicating the absence of detectable contaminating nucleic acids on the instrument and in the working area despite the high numbers of *C. auris* amplification experiments taking place on a regular basis. These data suggest that as long as standard operating procedures and proper decontamination protocols are followed, the risk of environmental contamination with *C. auris* is minimal.

**TABLE 5:** Results of 20 environmental swabs collected from our workspace when tested with the Diasorin *C. auris* IVD assay

Environmental location	Swabs tested	Direct IVD result	Mean IC CT
Interior (before testing)	5	Undetected (100%)	27.3
Interior (after testing)	5	Undetected (100%)	27.3
Exterior (before testing)	5	Undetected (100%)	27.3
Exterior (after testing)	5	Undetected (100%)	27.3

## DISCUSSION

This study is the first to assess the performance of the Diasorin Simplexa *C. auris* Direct assay (Diasorin *C. auris* IVD assay) in comparison to the current gold-standard method of culture followed by MALDI-TOF identification for the detection of *C. auris* colonization in clinical specimens. The Diasorin *C. auris* IVD assay demonstrated good agreement with a culture-based method. While there were two culture-negative specimens, it is notable that these specimens were both positive on each of the PCR-based methods examined in this study. The PCR-concordance but culture negativity could be explained by a higher sensitivity of PCR compared to culture in specimens with low numbers of organisms. Additionally, this study utilized retrospective frozen specimens, and freezing may have had a negative effect on the viability of the organisms. Lastly, our protocol for culture omitted the up to 5 day broth enrichment step outlined in the CDC procedure for isolation which may have reduced our culture sensitivity (17). Overall, the agreement between culture and the Diasorin *C. auris* IVD assay was high, suggesting this is a sensitive and specific method for the detection of *C. auris* colonization.

Additionally, this is the first study to compare the diagnostic accuracy of the Diasorin *C. auris* IVD assay to two lab-developed PCR tests in our lab, the DSQ *C. auris* LDT, and the Diasorin *C. auris* LDT. The Diasorin *C. auris* IVD assay demonstrated 100% clinical agreement with all *C. auris* positive samples tested on both LDTs examined in this study, providing great confidence that the Diasorin Simplexa *C. auris* Direct assay will provide results that are *at least* equivalent to other tests we already have used clinically at our institution.

Our experiments found a LoD of 702 CFU/mL, which is within the logarithmic range of the LoD described in the package insert (127 CFU/mL for Clade I, 260 CFU/ml for Clade IV)^13^. Given we still observed 85% detection of replicates at 233 CFU/mL, it is not unreasonable to hypothesize that our observed LoD could be lower if we had elected to test concentrations lower than 702 CFU/mL, and indeed, a sensitivity analysis of all of our data suggests it may be closer to 317 CFU/mL. More importantly, our previous LDT validations were performed with inactivated stock solutions quantified in copies/mL, which do not provide an assessment of the detection of live viable organisms. While the detection of both viable and non-viable organisms may be valuable in a screening assay, it is ultimately only viable organism that can lead to *C. auris* infection.

One of the critical features of any PCR-based test to detect *C. auris* is specificity, because a plethora of different microbes are known to colonize the skin including various yeasts, which can be misidentified as a number of different organisms when using traditional phenotypic methods for yeast identification. Inaccurate detection due to cross-reactivity could lead to unnecessary patient isolation, treatment errors, or misclassification of colonization status (18). Given the high microbial diversity in commonly surveilled anatomical regions like the axilla and groin, a high caliber of assay specificity is critical in clinical practice. In support of this need, we observed no off-target amplification from other *Candida* species or any of the phylogenetically related fungi and bacteria included in our panel of potentially cross-reactive organisms, indicating that the assay is not easily affected by off-target amplification events or organisms.

Given the antifungal drug resistance of *C. auris,* and its ability to easily colonize patients and patient care areas, there may exist concern in a clinical lab about contamination of lab space with *C. auris*. Additionally, labs accredited by the College of American Pathologists are required to monitor existing molecular testing for false positive results, including with wipe testing if necessary. Our lab routinely handles and grows *C. auris* and works with positive patient specimens. Environmental testing of the areas where we handle specimens for testing both before and after testing showed no evidence of *C. auris* positivity by PCR on the Diasorin *C. auris* IVD assay, affirming our lab practices.

As diagnostics for *C. auris* colonization continue to evolve, infection prevention practices evolve along with them and may dictate an ever-increasing need for rapid diagnostics. At our institution, we have experienced that need firsthand and despite our efforts to respond to the growing need to provide rapid diagnostics dictated by inpatient cohorting (19), disposition (20), and an ongoing need for contact precautions (21) that may affect the patient’s stay from both a cost perspective (22, 23) and a mental health perspective (24), we continue to find ourselves in need of an additional solution to provide rapid solutions for *C. auris* colonization diagnostics. Patients are often unable to transfer to an inpatient room from the Emergency Department or to discharge to a facility until the results of *C. auris* screening are back. We had settled upon the DSQ *C. auris* LDT on the open channel on the Roche Cobas 5800 platform because it allowed us to perform the assay twice daily in large, batched runs. Unfortunately, we have found we need to run the assay even more frequently to prevent delays in transitions of care. The Diasorin Simplexa Direct IVD (Diasorin *C. auris* IVD) assay allows for smaller run sizes in batches of 1–8, with turn-around times around 105 min. The Diasorin *C. auris* IVD assay offers an additional advantage over our previous Diasorin *C. auris* LDT with the absence of a separate pre-analytical step, and because it is FDA-approved allows for a far simpler verification and validation to onboard the assay in a clinical lab, even if multiple instruments are necessary.
